# Rat Hippocampal Neural Stem Cell Modulation Using PDGF, VEGF, PDGF/VEGF, and BDNF

**DOI:** 10.1155/2019/4978917

**Published:** 2019-03-18

**Authors:** Neus Gomila Pelegri, Catherine A. Gorrie, Jerran Santos

**Affiliations:** ^1^Neural Injury Research Unit, School of Life Sciences, University of Technology Sydney, NSW, Australia; ^2^Advanced Tissue Engineering and Drug Delivery Group, School of Life Sciences, University of Technology Sydney, NSW, Australia

## Abstract

Neural stem cells have become the focus of many studies as they have the potential to differentiate into all three neural lineages. This may be utilised to develop new and novel ways to treat neurological conditions such as spinal cord and brain injuries, especially if the stem cells can be modulated *in vivo* without additional invasive surgical procedures. This research is aimed at investigating the effects of the growth factors vascular endothelial growth factor, platelet-derived growth factor, brain-derived neurotrophic factor, and vascular endothelial growth factor/platelet-derived growth factor on hippocampal-derived neural stem cells. Cell growth and differentiation were assessed using immunohistochemistry and glutaminase enzyme assay. Cells were cultured for 14 days and treated with different growth factors at two different concentrations 20 ng/mL and 100 ng/mL. At 2 weeks, cells were fixed, and immunohistochemistry was conducted to determine cellular differentiation using antibodies against GFAP, nestin, OSP, and NF200. The cell medium supernatant was also collected during treatment to determine glutaminase levels secreted by the cells as an indicator of neural differentiation. VEGF/PDGF at 100 ng/mL had the greatest influence on cellular proliferation of HNSC, which also stained positively for nestin, OSP, and NF200. In comparison, HNSC in other treatments had poorer cell health and adhesion. HNSC in all treatment groups displayed some differentiation markers and morphology, but this is most significant in the 100 ng/ml VEGF/PDGF treatment. VEGF/PDGF combination produced the optimal effect on the HNSCs inducing the differentiation pathway exhibiting oligodendrocytic and neuronal markers. This is a promising finding that should be further investigated in the brain and spinal cord injury.

## 1. Introduction

It is well established that neurogenesis and gliogenesis occur in the adult nervous system [1], and in the past two decades, both neural progenitor cells (NPCs) and neural stem cells (NSCs) have been successfully isolated from the adult nervous system [2]. NSCs are found in the adult nervous system in the neurogenic regions like the hippocampus and the subventricular zone in the brain, as well as in the nonneurogenic regions in the subependymal layer lining the spinal cord central canal [2–5]. It is well documented that NPCs are upregulated after spinal cord injury in animals and that they respond to injury by proliferating, differentiating, and migrating to the site of injury, assumedly assisting in repair [6–8]. Consequently, these cells have become the focus of many studies as they are likely involved in the response to and an ideal therapeutic target in the development of therapies for neurological pathologies, such as spinal cord injury (SCI) and brain injury [2, 5, 9].

While neural cell transplantation is a promising treatment for central nervous system disorders [10, 11], it may be more advantageous to be able to manipulate endogenous neural progenitor cells or neural stem cells *in vivo*, without the need for invasive surgical procedures. Numerous growth factors are secreted by the nervous system and are naturally involved in cell proliferation and differentiation as part of development and in response to injury [12, 13]. In particular, platelet-derived growth factor (PDGF), brain-derived neurotrophic factor (BDNF), and vascular endothelial growth factor (VEGF) have been identified as growth factors that could be utilised for tissue repair as they have key roles in maintaining nervous system integrity. A recent study showed that NSCs proliferate *in vitro* in the presence of epidermal growth factor (EGF) and fibroblast growth factor (FGF) can be differentiated towards the oligodendrocytic lineage when cultured in PDGF [14]. On the other hand, BDNF has been shown to stimulate the differentiation, production, and survival of new neurons from the central nervous system derived NPCs [15–17]. VEGF has been shown to have a role in protecting neurospheres from hypoxia and serum withdrawal [18–20]. Promising research using *in vivo* models of rat spinal cord injury have shown that when PDGF and VEGF were infused in combination lesion size decreased, and animals showed functional recovery. However, when each of these growth factors was infused separately they showed detrimental effects [21–23].

We will use an *in vitro* model to examine the effects of PDGF and VEGF in isolation and in combination on the rat hippocampal neural stem cells (HNSCs). Cells grown with BDNF, B-27, and DMEM only will be included for comparison. Cell differentiation into oligodendrocytes, astrocytes, and neurons will be assessed using immunohistochemistry, immunofluorescence, and microscopy image analysis while neuronal cell differentiation will also be assessed using glutaminase enzyme secretion assay from medium supernatant.

## 2. Materials and Methods

### 2.1. Cell Culture Growth Factor Treatment

HNSCs previously isolated from the hippocampus of adult Sprague-Dawley rats by the Advance Tissue Engineering and Drug Delivery Group from the University of Technology Sydney (UTS) were utilised for the purpose of this project (UTS ACEC 2008-190A). HNSCs were isolated by exposing the skull of the rat, removing the skin and connective tissue from the cranium, and opening the skull from the base near the spine to the front of the skull above the brow in an inverted “V” shape allowing for easy removal of the whole brain. Once the brain was removed, it was coronally sectioned, and the isolated hippocampus was dissected into 2-3 mm^2^ sections. The sections were washed in 37°C sterile phosphate-buffered solution (PBS) and then incubated with 5 mL of 1.5 mg/mL collagenase solution to break down any collagen present in the connective tissue of the pieces. After discarding the digestion solution, the sections were placed in a T25 culture flask coated with poly-L-lysine (Nunc, MA, USA) with 5 mL of Neurobasal (Invitrogen, CA, USA) media supplemented with B-27® (Invitrogen, CA, USA). The flask was incubated at 37°C with 5% CO_2_ undisturbed for a week to allow cells to adhere.

Cells were revived from cryostorage under sterile conditions at passage 3 into one T25 flask with DMEM/F12+Glutamax media (Gibco, MA, USA) enriched with 10% heat-inactivated FBS (Sigma-Aldrich, MO, USA) and 1% penicillin+streptomycin (Gibco, MA, USA) and incubated at 37°C with 5% CO_2_. When the flasks reached 80%, confluence cells were expanded into a T75 flask using the same media and incubation conditions. Once the T75 reached 80% confluency, HNSCs were seeded into four 24-well plates at 20,000 cells/mL. Once cells reached 95 ± 2% confluence, treatment was started. HNSCs were treated under sterile conditions at all times using 0.5 mL of media+growth factors or just control media. The different growth factors/neurotrophic factors utilised were PDGF-BB (#PMG0044, Gibco, MA, USA), VEGF (#PHC9394, Gibco, MA, USA), and BDNF (#14-8366-62, Invitrogen, MA, USA). The growth factors were reconstituted following the manufacturer's instructions and were added fresh to the media at every change. The base media consisted of Dulbecco's modified Eagle's medium/nutrient mixture F12 (DMEM/F12)+Glutamax (Gibco, MA, USA) enriched with 1% penicillin+streptomycin (Gibco, MA, USA). Four different treatments were tested at two different concentrations in duplicate per plate: PDGF, VEGF, a combination of VEGF/PDGF, and BDNF at 20 ng/mL and 100 ng/mL together with a positive and negative control. The positive control was Neurobasal media (Invitrogen, CA, USA) supplemented with B-27® (Invitrogen, CA, USA), a commercially available medium supplement. The negative control consisted of the base media only DMEM/F12+Glutamax (Gibco, MA, USA) enriched with 10% heat-inactivated fetal bovine serum (FBS) (Sigma-Aldrich, MO, USA) and 1% penicillin+streptomycin (Gibco, MA, USA).

Cells were cultured for 2 weeks in the treatment groups, with media replaced and new growth factors/neurotrophic factors added every 84 h. Medium supernatants were collected and stored at -80°C for glutaminase quantification. At the end of the treatment, cell confluence was assessed and quantified to determine if cell numbers had increased or decreased.

### 2.2. Immunohistochemistry

Immunohistochemistry staining was conducted on four 24-well plates using the following primary antibodies: rabbit anti-glial fibrillary acidic protein (GFAP) (1 : 1000, Dako, Denmark), mouse anti-rat nestin (1 : 1000, Abcam, Cambridge, UK), rabbit anti-oligodendrocyte specific protein (OSP) (1 : 2000, Abcam, Cambridge, UK), and rabbit anti-neurofilament 200 (NF200) (1 : 200, Sigma-Aldrich, MO, USA) with goat anti-mouse AF568, goat anti-mouse AF488, or goat anti-rabbit AF488 (1 : 200, Invitrogen, CA, USA) secondary antibodies.

At day 14, cells were fixed inside the wells with 10% formalin for 30 minutes at room temperature under sterile conditions. After fixation, cells were washed using the phosphate-buffered saline with Triton X-100 at pH 7.4 (PBST). Then, 5% normal goat serum (NGS) in PBST was placed in the wells for 30 minutes as a blocking step. Primary antibodies were diluted in phosphate buffer with 5% NGS (PBG) and added to the relevant wells and incubated overnight at 4°C. Then, cells were washed with three changes of PBST and incubated with goat anti-mouse AF568 (1 : 200, Life Technologies, CA, USA), goat anti-mouse AF488 (Life Technologies, CA, USA), or goat anti-rabbit AF488 (1 : 200, Life Technologies, CA, USA) secondary antibody in PBG for 2 hours at room temperature. Wells were then washed with PBST and counterstained with Hoechst 1 : 5000 (Invitrogen, CA, USA) for 10 minutes to stain the nuclei and finally washed with PBST. Plates were imaged using IN Cell Analyzer 2200 high-content cellular analysis system (GE Healthcare Life Sciences, UK). Positive staining control cells were included in the GFAP, OSP, and NF200 staining runs. Glioblastoma U87MG cells were used as a GFAP positive staining control. Undifferentiated HNSCs were used as a positive staining control for nestin. PDGF-treated HNSCs were used as an OSP positive staining control, and neuroblastoma SHSY-5Y cells were used as a positive staining control for NF200. Both U87MG and SHSY-5Y cells were grown in separate plates to the experimental cells; however, the cells were stained in parallel with the experimental plates for each antibody and were fixed and stained following the same protocol as the experimental cells. U87MG cells were grown in a 24-well plate with DMEM/F12+Glutamax media (Gibco, MA, USA) enriched with 10% heat-inactivated FBS (Sigma-Aldrich, MO, USA) and 1% penicillin+streptomycin (Gibco, MA, USA) until confluence. SHSY-5Y were kindly grown and differentiated by Tara Nguyen. SHSY-5Y were grown in another 24-well plate with DMEM/F12+Glutamax media (Gibco, MA, USA) enriched with 10% heat-inactivated FBS (Sigma-Aldrich, MO, USA) and 1% penicillin+streptomycin (Gibco, MA, USA) for three days. After, the media were changed to DMEM/F12+Glutamax (Gibco, MA, USA) with hams media (Gibco, MA, USA) enriched with 15% heat-inactivated FBS (Sigma-Aldrich, MO, USA) and 1% penicillin+streptomycin (Gibco, MA, USA) with 50 nm of phorbol 12-myristate 13-acetate (Sigma-Aldrich, MO, USA) and 10 nm of nonessential amino acid to differentiate the cells.

### 2.3. Cell Imaging Analysis

Subsequent to immunohistochemistry staining, 10 randomized immunofluorescent images of each well were taken at 20x objective using the GE Healthcare Life Sciences–IN Cell analyzer 2200 high-content cellular analysis system.

Images were analysed with INcarta 1.4 GE Healthcare Life Sciences high-content cellular analysis system which was utilised to automate unbiased cell counting. A protocol was developed to select all stained nuclei, cells, and organelles, and then the images were manually checked to remove any poorly identified cells before counting. After analysis, representative images were selected, black balanced for figure arrangements.

### 2.4. Glutaminase Assay

A glutaminase assay was conducted to determine the amount of glutaminase enzyme secreted by the cells undergoing treatment in order to determine whether they exhibited properties expressed in neurons. Intracellular activities of glutaminase were measured by quantifying enzymatic interconversion of L-glutamine to L-glutamate using a colorimetric assay. NH_3_ levels are proportional to glutaminase, therefore, by quantifying NH_3_ levels, glutaminase levels can be determined. The cell medium supernatant was collected at each treatment change during cell culture and kept frozen at -80°C for glutaminase quantification.

(1) $$\begin{array}{c} \text{Glutamine}+{\text{H}}_{2}\text{O }\overset{\text{Glutaminase}}{\to }\text{Glutamate}+\text{N}{\text{H}}_{3} \end{array}$$

The assay was conducted by adding 50 *μ*L of the sample, 10 *μ*L of 0.1% *w*/*v* bromocresol purple indicator (Sigma-Aldrich, MO, USA), and 150 *μ*L of 100 mM L-glutamine glutamine (pH 8.5) (Sigma-Aldrich, MO, USA) to a 96-well plate. The absorbance was then read at 340 nm at different time points during 24 h incubation at 37°C in the TECAN infinity 200 plate reader.

No blank was utilised for the colorimetric assay as the fresh base medium (DMEM/F12+Glutamax+10% penicillin+streptomycin) is basic and the medium supernatant becomes acidic after being in contact with the cells.

### 2.5. Resazurin Redox Stress Assay

The assay was conducted on the cell supernatant to assess the stress levels on the cells caused by the different treatments after 14 days of treatment. The reagent is an oxidation-reduction indicator that changes colour in response to the chemical reduction of resazurin to resorufin. If resazurin is reduced, it indicates that the cells are experiencing a stress response therefore secreting an increased amount of reductive enzymes into the media.

The assay was conducted by adding 5 *μ*L of Alamar blue (Invitrogen) and 50 *μ*L of the test sample in a 96-well plate and incubated at 37°C for 2 h. The absorbance was then read at 570 nm using 600 nm as a reference wavelength at 37°C in the TECAN infinity 200 plate reader.

### 2.6. Statistical Analysis

GraphPad Prism software 7.03 was used to conduct the statistical tests. Two-way ANOVA with the Bonferroni post hoc tests was used to compare the means for two different concentrations and six different treatment groups. A *p* value of <0.05 was taken as an indicator of statistical significance for all ANOVA tests. All data is presented as mean and standard deviation.

## 3. Results

### 3.1. Cellular Counts

**Figure 1:**
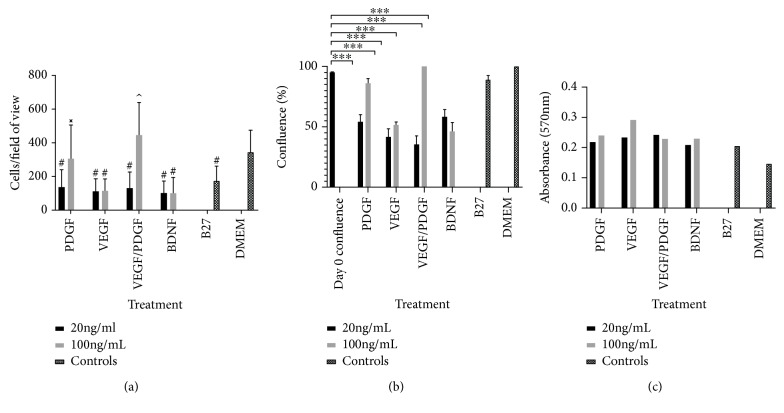
(a) Hippocampal neural stem cell counts per field of view in each growth factor treatment. Graph showing the number of cells per field of view for each growth factor treatment group at 20 ng/mL and 100 ng/mL vs. positive and negative controls after 14 days of treatment. ^#^Significantly different from DMEM (*p* < 0.001); ^x^significantly different from all treatments except for DMEM (*p* < 0.001); ^^^different from all treatments (*p* < 0.05). PDGF: platelet-derived growth factor; VEGF: vascular endothelial growth factor; BDNF: brain-derived neurotrophic factor; DMEM: Dulbecco's modified Eagle's medium. (b) % confluence of cells at day 0 vs. day 14. Graph showing cell confluence at day 0 vs. day 14 for each treatment group at 20 ng/mL and 100 mL and controls. Cell confluence significantly reduced after 14-day treatment in all treatments except in PDGF (100 ng/mL) and PDGF/VEGF (100 ng/mL); ^∗∗∗∗^*p* < 0.001. (c) Absorbance values at 570 nm for the resazurin redox stress assay at day 14 for each treatment.

Analysis of the mean cellular count of each treatment (Figure 1(a)) showed that there were 345.2 ± 130.4 cells per field of view in the DMEM (undifferentiated) control wells. This was reduced to 174.7 ± 86.9 in the B27 (differentiated) control wells (*p* < 0.001). The cell numbers for all treatments except the PDGF 100 ng/mL and VEGF/PDGF 100 ng/mL were lower compared to the DMEM control (*p* < 0.001). However, the VEGF/PDGF 100 ng/mL cell counts of 445.8 ± 194.2 were the only treatment to increase cell numbers from all other treatments (*p* < 0.05) including the DMEM levels. There were no observable differences noted in any of the other parameters measured, i.e., cell length, cell area, nuclei area, nuclei length, and cell elongation, and these were not examined further in the study.

Cell confluence (Figure 1(b)) significantly decreased (*p* < 0.001) in all treatments except in PDGF 100, VEGF/PDGF 100, and the positive (B27) and negative controls (DMEM only). PDGF 100 and VEGF/PDGF were the only treatments that the confluence did not decrease between the start of the treatment and the end of the treatment. The stress assay (Figure 1(c)) correlates with cell numbers decreasing. When there is a decrease in cells, there is an increase in the resazurin reduction indicative of cellular stress.

### 3.2. Cellular Differentiation

#### 3.2.1. GFAP Expression

GFAP is a type III intermediate filament commonly found in the cytosol of mature astrocytes and in NSCs isolated from a mammalian adult brain [24, 25]. In this project, it will be used as a marker for both mature astrocytes and immature HNSCs.

**Figure 2:**
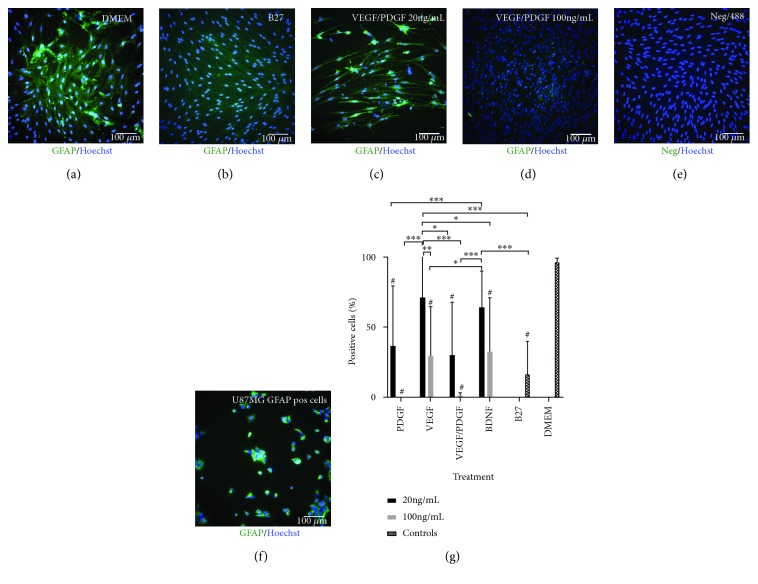
GFAP expression after 14-day treatment. PDGF/VEGF at 100 ng/mL has the lowest expression of GFAP after treatment. (a) DMEM undifferentiated control cells. (b) B27-positive differentiating control. (c) HNSC cultured with base media and 20 ng/mL of VEGF/PDGF. (d) HNSC cultured with base media and 100 ng/mL of VEGF/PDGF. (e) Negative staining control. Primary antibody omitted. (f) U87MG-positive staining control. All cells stained with a primary GFAP antibody and an AF488 secondary antibody (green). Nuclei stained with a Hoechst antibody (blue). (g) GFAP-positive cell percentage. Graph showing the percentage of GFAP-positive cells for each growth factor treatment group at 20 ng/mL and 100 ng/mL vs. positive and negative controls after 14 days of treatment. *p* < 0.001; ^∗^*p* < 0.05; ^∗∗^*p* < 0.01; and ^∗∗∗^*p* < 0.001; ^#^significantly different from DMEM.

GFAP expression analysis (Figure 2) shows that 96.3 ± 2.9% of the cells in the DMEM (undifferentiated) control group stained positive for GFAP. Furthermore, GFAP expression has significantly reduced in all treatments (*p* < 0.001) compared to the DMEM (undifferentiated) control except in the VEGF 20 ng/mL treatment where 71.1 ± 37.1% of the cells stained positive for GFAP. PDGF 100 ng/mL had no GFAP expression and VEGF/PDGF 100 ng/mL only had 1.2 ± 2% of cells staining positive for GFAP. B27 (differentiated) control group presented with 16.4 ± 23.4% of GFAP positive cells.

#### 3.2.2. Nestin Expression

Nestin is a type IV intermediate filament found in the cytosol of neural progenitor cells and in this project, will serve as a marker for those cells [26, 27].

**Figure 3:**
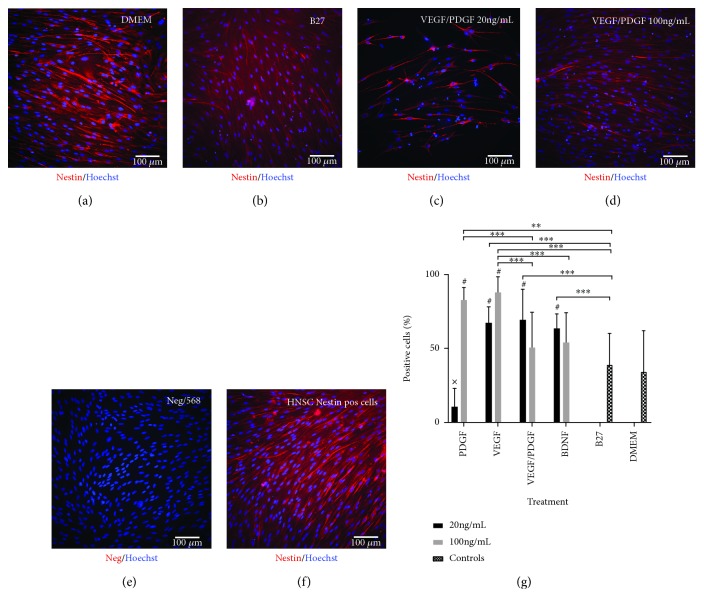
Nestin expression after 14-day treatment. (a) DMEM undifferentiated control cells. (b) B27-positive differentiating control. (c) HNSC cultured with base media and 20 ng/mL of VEGF/PDGF. (d) HNSC cultured with base media and 100 ng/mL of VEGF/PDGF. (e) Negative staining control. Primary antibody omitted. (f) HNSC positive staining control. All cells stained with a primary nestin antibody and an AF568 secondary antibody (red). Nuclei stained with a Hoechst antibody (blue). (g) Nestin-positive cell percentage. Graph showing the percentage of nestin-positive cells for each growth factor treatment group at 20 ng/mL and 100 ng/mL vs. positive and negative controls after 14 days of treatment. *p* < 0.001; ^∗^*p* < 0.05; ^∗∗^*p* < 0.01; and ^∗∗∗^*p* < 0.001; ^#^significantly different from DMEM (*p* < 0.01); ^x^significantly different from all treatments except for DMEM (*p* < 0.001).

Nestin expression analysis (Figure 3) shows that in the DMEM (undifferentiated) control group only 34.2 ± 27.8% of cells stained positive for nestin. Nestin expression is significantly higher after all treatments compared to the DMEM (undifferentiated) control wells (*p* < 0.01) except for VEGF/PDGF 100 ng/mL and B27 control. VEGF/PDGF 100 ng/mL presented with 50.5 ± 23.9% of cells staining positive for nestin. In the B27 control group only 39.0 ± 21.2% of cells stained positive for nestin. On the other hand, PDGF 20 ng/mL shows the lowest expression of nestin with only 10.5 ± 12.6% of cells stained positive for nestin being significantly lower to all treatments except from DMEM control (*p* < 0.001). VEGF 100 ng/mL has the highest expression of nestin with 87.9 ± 10.5% of the cells staining positive followed by PDGF 100 ng/mL with 82.6 ± 8.7% of cells stained positive for nestin.

#### 3.2.3. OSP Expression

OSP is an oligodendrocyte specific surface protein found on mature oligodendrocytes [28, 29]. In this project, it will be used to visualise those cells.

**Figure 4:**
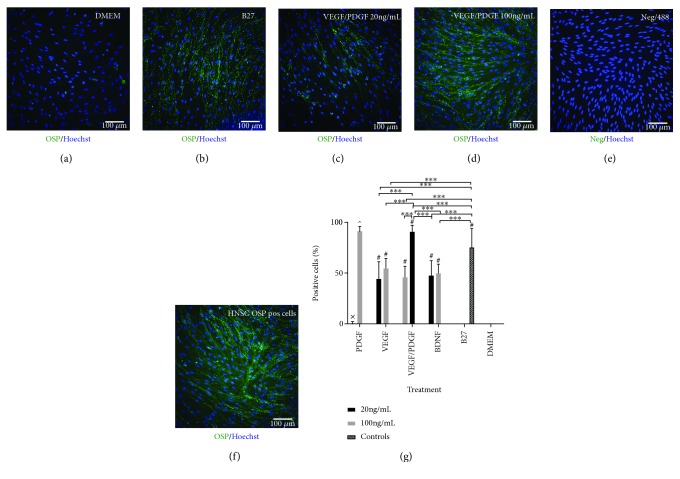
(a–f) OSP expression after 14-day treatment. PDGF/VEGF at 100 ng/mL has the highest OSP expression after treatment. (a) DMEM undifferentiated control cells. (b) B27-positive differentiating control. (c) HNSC cultured with base media and 20 ng/mL of VEGF/PDGF. (d) HNSC cultured with base media and 100 ng/mL of VEGF/PDGF. (e) Negative staining control. Primary antibody omitted. (f) HNSC positive staining control. All cells stained with a primary GFAP antibody and an AF488 secondary antibody (green). Nuclei stained with a Hoechst antibody (blue). (g) OSP-positive cell percentage. Graph showing the percentage of OSP-positive cells for each growth factor treatment group at 20 ng/mL and 100 ng/mL vs. positive and negative controls after 14 days of treatment. ^∗∗∗^*p* < 0.001; ^#^significantly different from DMEM (*p* < 0.001); ^x^significantly different from all treatments except for DMEM (*p* < 0.001); ^^^significantly different from all treatments except for VEGF/PDGF (100 ng/mL) (*p* < 0.001).

OSP expression analysis (Figure 4) shows no OSP expression in the cells cultured in DMEM only with all treatments except PDGF 20 ng/mL showing a significantly higher OSP expression than DMEM control (*p* < 0.001). In the PDGF 20 ng/mL treatment only 0.5 ± 1.6% of cells stained positive for OSP. On the other hand, PDGF/VEGF 100 ng/mL shows the highest expression of OSP compared to all treatments with 90.8 ± 6.4% of cells staining positive for OSP being significantly higher than the B27 control group (*p* < 0.001) with 75.5 ± 18.3% of cells stained positive for OSP. PDGF 100 ng/mL is also significantly higher all treatments except VEGF/PDGF 100 ng/mL (*p* < 0.001) with 91.3 ± 4.6% of cells stained positive for OSP.

#### 3.2.4. NF200 Expression

Neurofilament 200 is an intermediate found in the cytoskeleton supporting the axon cytoplasm of mature neurons [30].

**Figure 5:**
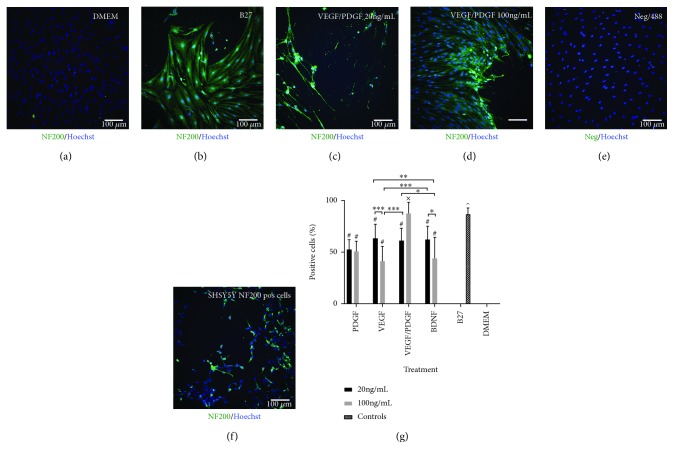
(a–f) NF200 expression after 14-day treatment. PDGF/VEGF at 100 ng/mL has expression levels of NF200 similar to those of B27. (a) DMEM undifferentiated control cells. (b) B27-positive differentiating control. (c) HNSC cultured with base media and 20 ng/mL of VEGF/PDGF. (d) HNSC cultured with base media and 100 ng/mL of VEGF/PDGF. (e) Negative staining control. Primary antibody omitted. (f) SHSY-5Y positive staining control. All cells stained with a primary GFAP antibody and an AF488 secondary antibody (green). Nuclei stained with a Hoechst antibody (blue). (g) NF200-positive cell percentage. Graph showing the percentage of NF200-positive cells for each growth factor treatment group at 20 ng/mL and 100 ng/mL vs. positive and negative controls after 14 days of treatment. *p* < 0.001; ^∗^*p* < 0.05; ^∗∗^*p* < 0.01; and ^∗∗∗^*p* < 0.001; ^#^significantly different from DMEM (*p* value < 0.001); ^^^significantly different from all treatments except for VEGF/PDGF (100 ng/mL) (*p* < 0.001); ^x^significantly different from all treatments except for B27.

NF200 expression analysis (Figure 5) has shown that all treatments have significantly higher NF200 expression compared to DMEM control that shows no expression (*p* < 0.001). B27 shows the highest expression of NF200 being significantly higher from all treatments except from VEGF/PDGF 100 ng/mL (*p* < 0.001) with 86.9 ± 5.9% of cells staining positive for NF200. In the VEGF/PDGF 100 ng/mL treatment 87.5 ± 10.84% of the cells stained positive for NF200 being significantly higher to all treatments except from B27 (*p* < 0.001).

### 3.3. Glutaminase Assay

**Figure 6:**
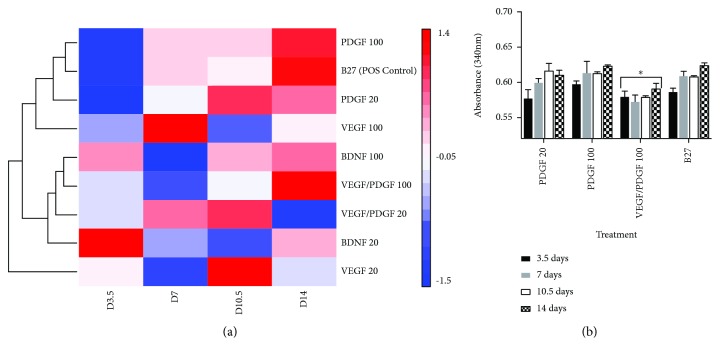
Glutaminase secretion levels after 14-day treatment. (a) Euclidean test grouping the treatments across all time points into hierarchical clusters of similarity according to the concentration of glutaminase secreted. Heat map showing the log_10_ of absorbance clustered in groups according to the highest glutaminase expression. (b) Graph showing the treatment groups that showed the same trend in glutaminase increase during the 14-day treatment. ^∗^significantly different from all other treatments (*p* < 0.05).

Glutaminase assay analysis showed that the cells treated with PDGF 100 ng/mL, B27, PDGF 20 ng/mL, and VEGF/PDGF 100 ng/mL secreted the highest amounts of glutaminase enzyme during the 14-day treatment. The Euclidean test analysis that was performed has grouped the treatments across all time points into hierarchical clusters of similarity according to the concentration of glutaminase secreted (Figure 6(a)). As such, PDGF 100 ng/mL had the closest glutaminase secretion profile to the B27 positive control, followed by VEGF/PDGF 100 ng/mL. The treatments that showed increased glutaminase were then graphed with increasing absorbance values to show changes over the 14-day period (Figure 6(b)).

## 4. Discussion

We observed that PDGF alone and the combination of VEGF/PDGF at higher concentration showed the best outcomes for cellular health, proliferation, and differentiation of HNSCs relative to other growth factors and combinations tested. As expected, the DMEM (undifferentiated) negative control remained undifferentiated during the treatment process expressing mainly GFAP immunohistochemistry marker known to be expressed in immature neural cells [24, 25] and some nestin expression indicating that the pool of cells utilised had some more committed cells present [26, 27, 31]. B27 (differentiating) control showed signs of neural differentiation expressing lower levels of GFAP and increased levels of nestin compared to the DMEM (undifferentiated) control and high expression levels of OSP and NF200 markers indicating cellular differentiation towards the oligodendrocyte and neuronal pathway. Cells in most treatments were also found to coexpress nestin, OSP, and NF200. It has been previously documented that astrocytes, oligodendrocytes, and neurons can also express nestin [32–34]. Overall, the immunohistochemistry results suggest that the cells reached an intermediate stage of cellular differentiation which is supported by the glutaminase results that showed that PDGF at both concentrations and VEGF/PDGF at high concentration displayed the higher amount of glutaminase secretion indicative of neural differentiation after 14-day treatment. Glutaminase is an enzyme that is mainly responsible for converting glutamine to glutamate which serves as a key excitatory neurotransmitter in the brain [35]. Moreover, glutamine transporter is more predominant in neurons than in other neural cells and glutaminase is upregulated during neuronal differentiation making it an ideal marker to determine neural stem cell maturation towards the neuronal lineage [35–37].

### 4.1. BDNF Increases Neural Differentiation but Does Not Increase Cellular Numbers

BDNF treatment stimulated HNSC differentiation, but it did not show an increase in cell number at 14 days. HNSCs after 14 days of treatment with BDNF at both concentrations displayed NF200 and OSP marker expressions indicative of neural differentiation. BDNF was included in this study as it has been widely shown to promote neural differentiation and neuronal survival [15–17, 38]. BDNF is known to have critical functions in promoting survival, proliferation, and differentiation of NSCs, but its downstream mechanisms are not yet fully understood [39].

All known BDNF receptors are present in NSCs. More specifically, BDNF exerts its proliferative effects on NSCs through the truncated tropomyosin receptor kinase B (t-TRKB) [40]. It has been suggested that BDNF stimulates NSC proliferation, and it effectively enhances cell commitment to neuronal and oligodendrocytic fates via the MAPK pathway that in turn triggers the Wnt/beta-catenin signaling pathway, a pathway involved in embryonic neuronal development. Similarly, BDNF also protects against neurotoxic-induced NSC apoptosis via binding to the tropomyosin receptor kinase B (TRKB) and activating PI3K (cell cycle) and MAPK pathways [39].

On the other hand, some *in vivo* studies have observed that BDNF infusion reduced neurogenesis [41, 42], and it has been shown that both BDNF and glial-derived neurotrophic factor are required for the survival of neurons *in vivo* [43]. This could explain why an increase in cell numbers was not apparent in our study when cells were treated with BDNF alone.

### 4.2. VEGF Does Not Induce Cellular Proliferation, but It Affects Neural Stem Cell Differentiation

The results showed that VEGF alone did not favour cell proliferation and cellular health; however, immunohistochemistry results showed that VEGF did affect cell differentiation. GFAP levels dropped after 14-day treatment in the VEGF at high concentrations, and OSP and NF200 marker expressions increased, suggesting that VEGF did push the HNSCs to differentiate. Therefore, low proliferation could be due to cellular maturation.

VEGF has been shown to stimulate adult neural stem cells *in vitro*; however, the treatment utilised VEGF after growing the neural stem cells into neurospheres and subsequent treatment with VEGF [44]. It is possible that the VEGF treatment was not conducted for long enough in the current study for the cells to show full effects or that the cells would require complementary growth factors to initiate the change before VEGF displays its effects.

The VEGF and VEGF receptors are present in NSCs and exert their effects via downstream signaling pathways: the MEK-MAPK pathway (proliferation and migration), the PIK3-AKT pathway (survival), and the SRC-eNOS pathway (permeability) [45]. It has also been shown that VEGF *in vivo* and *in vitro* stimulates cellular proliferation in the brain [18, 19] and induces neurite maturation and neuronal growth in primary CNS neuronal cultures mediated by VEGF receptor 2 (VEGFR2) via the MAPK pathway [20].

Furthermore, NSCs express VEGF receptor 3 (VEGFR3) and its ligand VEGF-C which are responsible for the activation of quiescent NSCs. VEGFR3 activates the ERK/AKT signaling pathway that promotes NSC activation and differentiation into NPCs in rodents [46]. This would explain the high levels of nestin expression after treatment. On the other hand, VEGF receptor 2 (VEGFR2) regulates the promotion of axonal outgrowth and neuronal survival and increases the number and length of neurites when activated by VEGF by activating the MAPK and PI3K/Akt pathways [47].

SCI animal models also showed that VEGF increased tissue in the lesion site and vascular density and it decreased apoptosis following SCI [23]. Contrary to this, other experiments using VEGF and PDGF infusion in rat spinal cord injury models showed that when VEGF and PDGF were administered alone they aggravated the injury and increased the injury cavity size [21]. This could be due to the fact that VEGF is a vascular permeability factor that at high concentrations can compromise CNS homeostasis by disrupting the blood brain barrier (BBB) [45] which may explain the contradictory findings above.

### 4.3. PDGF Alone Induces Neural Stem Cell Differentiation and an Increase in Cell Numbers

Proliferation decreased marginally in the PDGF treatment at a high concentration compared to the DMEM undifferentiated control implying that the PDGF alone is able to positively stimulate cell proliferation. Furthermore, cells under the influence of PDGF at a high concentration did not express GFAP but showed high expressions of nestin, NF200, and OSP markers as well as high extracellular secretion of glutaminase indicating cellular differentiation down the neuronal and oligodendrocytic lineage. PDGF is known to play a role during neuronal differentiation [48] and in oligodendrocyte maturation [14] and it also has neuroprotective effects in animal models with neuronal injury [49]. Moreover, PDGF is required for healthy oligodendrocyte development and normal myelination in the spinal cord and cerebellum and has an essential role in wound healing [50]. However, there is no evidence that VEGF and BDNF have an effect on oligodendrocyte maturation.

PDGF-BB was used in the current study because that is what was used successfully in an *in vivo* combination treatment of PDGF and VEGF in rat spinal cord injury models [22]. There are in fact four PDGF ligands and PDGF receptors that are essential proteins expressed in neural stem/progenitor cells, neurons, astrocytes, and oligodendrocytes. PDGF-BB can activate both PDGF alpha-receptor (PDGFR*α*) and PDGF beta-receptor (PDGFR*β*) [50, 51]. The activation of PDGFR*α* controls oligodendrocyte progenitor cell (OCP) proliferation, migration, survival, and maturation. The activation of PDGFR*α* is dependent on the activation of both PI3K and PLC*γ* pathways. These pathways are concentration-dependent; to induce migration, only the PI3K pathway activation is required with low ligand concentration, whereas for proliferation and maturation, both PI3K and PLCγ pathway activations are required with high ligand concentration. This means that the strength of the signal received dictates the pathway activation during OCP maturation [52]. Furthermore, PDGF produces several rounds of divisions before pushing the cells to differentiate into oligodendrocytes [53] explaining why the cells under the effect of PDGF at high concentration continued proliferating. Additionally, PDGFR*β* regulates differentiation towards the neuronal lineage [51]. The results obtained suggest that in these experiments PDGF-BB activated both PDGFR*α* and PDGFR*β* receptors in the HNSCs pushing the cells down the neuronal and oligodendrocytic differentiation pathways.

### 4.4. VEGF and PDGF Have Adding Effects on the Cells and Induce Cell Number Increase and Cellular Differentiation

Cell proliferation in all growth factor treatments dropped when compared to the DMEM control except in the PDGF/VEGF treatment at a high concentration in which proliferation increased in comparison to the DMEM (undifferentiated) control. Furthermore, VEGF/PDGF at a high concentration looked the healthiest of all treatments in terms of cellular adhesion and confluence, compared to the DMEM (undifferentiated) control. Regarding cellular morphology, cells under the influence of VEGF/PDGF showed high numbers of bipolar cells together with some elongated spindle-shaped cells. Immunohistochemistry results on VEGF/PDGF at a high concentration showed that the growth factor treatment pushed the cells down the oligodendrocytic and neuronal differentiation pathway as no GFAP was expressed after the 14-day treatment, and OSP and NF200 increased at similar levels to the B27 (differentiating) control. Moreover, glutaminase levels in VEGF/PDGF 100 ng/mL increased during treatment indicating cellular maturation. However, the total increase in glutaminase is less than in the other treatments. It is believed that it is due to the direct effect of VEGF/PDGF at 100 ng/mL.

The fact that both VEGF and PDGF alone had positive effects and that the growth factor combination of VEGF/PDGF at high concentrations displayed the best outcomes suggest that the growth factors complement and favour each other showing synergistic effects. It has been previously observed that when the VEGF/PDGF growth factor combination was injected in *in vivo* rat SCI models, the treatment combination showed positive recovery outcomes [21, 22]. These results indicate that this particular growth factor treatment does have a positive effect on the undifferentiated HNSCs.

The positive outcomes of VEGF/PDGF growth factor combination might be due to the effects of the growth factors in their receptors as VEGF and PDGF genes and polypeptides belong to a functionally and structurally related family. The Pdgfa gene is known to encode for a protein found in both VEGF and PDGF. VEGF and PDGF also interact with similar receptors to activate neural differentiation [50, 51]. PDGF acts via tyrosine kinase PDGFR*α* and PDGFR*β* receptors while VEGF acts through different but structurally related subfamily of receptor tyrosine kinase (RTK) VEGF1, VEGF2, and VEGF3.

The results found that VEGF/PDGF combination at a high concentration mostly pushed HNSCs down the oligodendrocyte pathway. However, results on PDGF at both concentrations revealed that the cells under that treatment alone differentiated towards both neuronal and oligodendrocyte differentiation pathways. PDGF-BB binds to both PDGFR*α* and PDGFR*β* to induce differentiation towards oligodendrocytes and neurons, respectively. On the other hand, VEGF-A is structurally similar to PDGF-CC, and it is capable of activating PDGFR*α* [50, 51]. Therefore, it is likely that in this experiment PDGF-BB activated both PDGFR*α* and PDGFR*β* and VEGF activated PDGFR*α* when in combination with PDGF.

Additionally, VEGF has also shown to protect cultured cerebral neurons or hippocampal neurons during hypoxia or serum withdrawal and also protects them against glutamate or NMDA toxicity [54]. Therefore, it is also possible that in these experiments, VEGF protected HNSCs against serum starvation, and PDGF pushed the cells down the oligodendrocytic differentiation pathway.

## 5. Conclusions

It was previously reported that the administration of the VEGF/PDGF growth factor combination improved outcomes following SCI in rat models [21, 22]. The current study showed that the combined VEGF/PDGF treatment resulted in high levels of hippocampal neural stem cell differentiation towards oligodendrocytic and neuronal cell lineages. This is a promising finding as neural stem cell activation and modulation have the potential to be developed into a novel treatment for neurological conditions such as spinal cord and brain injuries. It will be even more beneficial if stem cells can be modulated *in vivo* without the need for additional invasive surgical procedures. Therefore, these findings provide proof of concept and suggest that this line of research should be explored further. We will now include these growth factor combinations in our trial using the spinal cord neural progenitor cells and other neural stem cell populations.

## Data Availability

The data used to support the findings of this study are available from the corresponding author upon request.
